# “People have options”: a qualitative study of experiences and influences of PrEP choice among women in South Africa

**DOI:** 10.1002/jia2.26462

**Published:** 2025-07-02

**Authors:** Siphokazi Dada, Faith Mary Musvipwa, Fatima Abegail Cholo, Melanie Pleaner, Alison Kutywayo, Vusile Butler, Catherine Elizabeth Martin, Saiqa Mullick

**Affiliations:** ^1^ Wits RHI University of the Witwatersrand Johannesburg South Africa

**Keywords:** choice counselling, dapivirine vaginal ring, HIV prevention, pre‐exposure prophylaxis, South Africa, women

## Abstract

**Introduction:**

Long‐acting injectable cabotegravir and dapivirine vaginal ring were recently introduced in South Africa through demonstration projects, providing additional HIV prevention options to oral pre‐exposure prophylaxis (PrEP). PrEP choice marks a pivotal moment in HIV prevention, potentially increasing PrEP uptake and use by offering women a choice of methods best suited to their individual needs. Understanding experiences of PrEP choice in real‐world settings is critical to informing the introduction of PrEP choice at scale. This study, embedded within an implementation science study introducing new PrEP methods, explores women's experiences of PrEP choice counselling, and factors influencing PrEP choice.

**Methods:**

Between April and May 2024, we conducted 12 focus group discussions with a sub‐set of 126 women (18–57 years) enrolled in a parent cohort study delivering PrEP choice at six fixed and three roving mobile clinics in three areas of South Africa. Cohort participants are HIV‐negative men and women ≥ 15 years interested in HIV prevention services. At the time of data collection, non‐pregnant and lactating women ≥ 18 years enrolled in the cohort were provided PrEP choice counselling and offered a choice of oral PrEP or dapivirine vaginal ring. Discussions explored women's experiences of PrEP choice counselling and influences of PrEP choice. They were audio recorded, transcribed verbatim and translated into English for thematic analysis.

**Results:**

Women reported positive choice counselling experiences, particularly when it was supplemented by pamphlets, demonstration rings and pelvic models. Participants valued learning about alternative PrEP methods and appreciated friendly healthcare providers who answered their questions. Privacy and emotional support were also crucial. However, negative experiences included the use of complex scientific jargon and insufficient information on PrEP's potential drawbacks. PrEP choices were mainly influenced by concerns about intravaginal products, distrust of new methods, prior oral PrEP experiences, and partner and family opinions.

**Conclusions:**

These findings will guide strategies for PrEP delivery, emphasizing provider training, effective counselling tools and tailored communication. Women valued clear, jargon‐free information, visual aids and a welcoming environment, which supported open dialogue. The influence of prior oral PrEP experiences on PrEP choice highlights the need for counselling that addresses specific concerns and preferences.

## 1 INTRODUCTION

Globally, despite declining HIV incidence, approximately 1.3 million new acquisitions occurred in 2023, most among young women in sub‐Saharan Africa, highlighting the ongoing need for effective prevention methods [1]. Daily oral pre‐exposure prophylaxis (oral PrEP) has been included in combination HIV prevention in South Africa since 2016 [2]. In 2021, the WHO recommended the dapivirine vaginal ring (ring) [3] and in 2022 long‐acting injectable cabotegravir (CAB‐LA), expanding HIV prevention options [4]. Both the ring and CAB‐LA were approved in South Africa in 2022 and are available at research and pilot sites. These methods hold the potential to overcome challenges with daily oral PrEP and increase effective PrEP use [5]. Emerging evidence further suggests that offering various PrEP methods can enhance prevention coverage and lower HIV incidence [6]. We have learnt from contraception that expanded choice coupled with informed‐choice counselling, results in improved utilization and continuation [7, 8, 9]. While previous PrEP studies offered women an opportunity to use different methods [10], none provided PrEP choice counselling. There remains a gap in our understanding of women's experiences with PrEP choice counselling, how this supports informed decision‐making, what influences women's PrEP choice and how these are experienced in routine, integrated HIV prevention services.

The delivery of PrEP choice requires more than just the availability of PrEP methods. Access to quality services, comprehensive information, trained healthcare providers (HCPs) and effective counselling are critical [11]. Choice counselling bridges the gap between PrEP method availability and informed decision‐making [12]. It is a dynamic, two‐way discussion between clients and HCPs, assisting clients to determine the best method choice for them, considering their lifestyle, preferences and concerns [12]. It is also important to recognize that the process of informed choice begins before individuals arrive at a health facility. Community engagement, social media and information provided by peers play a vital role in raising awareness and educating women about prevention methods [13].

To contextualize choice counselling in PrEP and inform approaches to delivering choice at scale, it is crucial to understand the real‐world experiences of women, and the influences of their decisions. The aim of this study was to explore women's experiences of PrEP choice counselling and the factors influencing their method choice within primary care services in South Africa.

## 2 METHODS

### 2.1 Parent study

Project PrEP, a Unitaid‐funded implementation science study, has supported the introduction of oral PrEP [14], and is currently introducing new PrEP methods within primary healthcare services in South Africa. The study is implemented in four geographical areas, each comprising two fixed public healthcare clinics and a linked, roving mobile clinic: a peri‐urban area in Gqeberha, Eastern Cape; the rural town of Mthatha, Eastern Cape, an urban area of eThekwini, KwaZulu‐Natal and a peri‐urban area of Tshwane, Gauteng. Since August 2023, sites in three of the four areas (i.e. Gqeberha, Mthatha and Tshwane) have been offering the ring in addition to oral PrEP, with CAB‐LA introduced in April 2024. PrEP method choice is offered to participants enrolled in parent cohort, following national guidelines [15]. Cohort participants are HIV negative, ≥ 15 years and interested in HIV prevention services, including PrEP naïve, prior or current PrEP users. Information on PrEP methods is provided through pamphlets, with generic PrEP information available online and through social media [16]. Initially, and at the time of data collection for this study, pamphlets contained information on the ring and oral PrEP. These were updated to include information on CAB‐LA after its introduction. PrEP choice counselling is provided by trained nurses, using job aids to guide the session and ensure consistent delivery of information. These include visual guides, checklists and step‐by‐step instructions for each method. Demonstration of correct ring insertion techniques is provided, and participants are given the opportunity to insert the ring themselves to build confidence. At follow‐up visits, participants are given the option of taking up to three rings, which they can self‐insert monthly at home. Despite the recent availability of CAB‐LA at some study sites at the time of data collection, this study aimed to explore early experiences of PrEP choice and experiences of PrEP ring choice and use, and thus data collection did not explore experiences of CAB‐LA choice and use.

### 2.2 Study design, recruitment and study population

This study employed a phenomenological and exploratory approach utilizing focus group discussions (FGDs), to capture participants’ lived experiences and perspectives. A subset of women ≥ 18 years, currently or previously using oral PrEP or the ring, and enrolled in the parent cohort study at implementing sites in Gauteng and the Eastern Cape were purposively selected and invited to participate. Recruitment was conducted by site‐based fieldworkers during clinic visits, or telephonically, with participants grouped according to the PrEP method they were using or had used. Informed consent was obtained, and basic demographic information was collected prior to FGDs commencing. Our study followed the Consolidated Criteria for Reporting Qualitative Research (COREQ) [17].

### 2.3 Data collection

FGDs were conducted between April and May 2024, approximately 9 months after the introduction of the ring at sites. Twelve single face‐to‐face FGDs were held with 126 women: six with oral PrEP users and six with ring users, ranging from 6 to 19 participants per group. FGDs were conducted in a local language (Setswana and isiXhosa), in a private room either at the facility or a nearby community‐based organization, by two female researchers, trained and experienced in qualitative data collection (FAC and SD). A semi‐structured guide, developed by the researchers, was used to direct the discussion, exploring experiences of choice counselling and factors influencing PrEP choice decision‐making. FGDs lasted 59 minutes to 2 hours and were audio recorded.

### 2.4 Data analysis

Audio recordings were transcribed verbatim, translated into English and cross‐checked by the study team to ensure accuracy before analysis. The analysis team included four researchers (SD, FAC, FMM and AK) and a Technical Specialist (CEM). We focused on capturing diverse perspectives, which resulted in a broad range of experiences rather than reaching data saturation on a single aspect. We used the Socio‐Ecological Model (SEM) framework to structure the analysis, applying it to the component of the results which explored the factors influencing women's PrEP method choice. However, the societal level was not explored because factors influencing PrEP choice at this level did not emerge from the data. Women's experiences with PrEP counselling were grouped into positive and negative experiences. Data analysis was inductive and followed six phases of thematic analysis [18] and NVivo 14 was used for coding. Two researchers (FAC and FMM) independently coded three transcripts using open coding, compared codes to ensure consistency and developed a preliminary coding framework. This was reviewed by the team, revised and the coding framework finalized, after which the remaining transcripts were analysed.

### 2.5 Theoretical framework

The SEM [19] framework used in this analysis is useful in understanding the factors that influence health behaviour, including HIV prevention [19] and has been used in other studies to contextualize facilitators and barriers to uptake and use of HIV prevention methods [20]. The SEM illustrates five interdependent levels of influence namely (i) individual: focusing on personal knowledge, attitudes and beliefs; (ii) interpersonal/relationship: focusing on social networks’ influences including family, friends and intimate partners; (iii) health system: focusing on healthcare services, accessibility and the quality of counselling provided by HCPs; (iv) community: focusing on norms, stigma and collective beliefs; and (v) societal: focusing on cultural norms, policies and laws.

### 2.6 Ethical considerations

The study was approved by the Human Research Ethics Committee at the University of the Witwatersrand (220604) and the World Health Organization (WHO) Ethics Research Committee (ERC.0003784). All participants provided written informed consent for participation and audio recording of FGDs and were reimbursed R300 (17.5USD) for their time and travel costs.

## 3 RESULTS

Of the 126 participants, the majority (61.1%) were < 25 years, almost all were in a relationship (92.1%), the majority had completed high school (62.7%) and 80.9% were unemployed (Table 1). By design, there was an approximately equal split of current oral PrEP and ring users, although 84.1% had experience using oral PrEP and 63.2% had used the ring.

**Table 1:** Characteristics of the study participants who were provided a choice of oral or ring pre‐exposure prophylaxis (PrEP)

Characteristics	Total (*N* = 126) 100%
Age group	
18−24 years	77 (61.1%)
≥25 years	49 (38.9%)
Median age (IQR [p25–p75]) (years)	22.5 (21−26)
Highest level of education completed	
Some secondary education	22 (17.5%)
Secondary (Grade 12)	79 (62.7%)
Tertiary or further education training	25 (19.8%)
Employment status	
Employed	24 (19.17%)
Unemployed	102 (80.9%)
Relationship status	
In a relationship	116 (92.1%)
Single, no partner	10 (7.9%)
PrEP method currently using	
Oral PrEP	63 (50.0%)
Ring	55 (43.7%)
Ever used oral PrEP	106 (84.1%)
Ever used the ring	67 (63.2%)

Abbreviations: IQR, interquartile range; PrEP, pre‐exposure prophylaxis.

We categorized participants’ experiences into two main concepts—first, experiences of PrEP choice counselling and second, factors influencing PrEP method choice.

### 3.1 Experiences of PrEP choice counselling

We identified the following positive themes in relation to women's experiences:

- **Receiving comprehensive information on PrEP methods**

Participants reported receiving comprehensive information on both PrEP methods during choice counselling, including information on how the methods work, their administration and potential side effects; sufficient for making an informed decision about their method choice.

“The information was enough because I remember the nurse said if you are unable to remove the ring yourself after 28 days you can come back to the clinic; she will help me remove it…” (Mthatha, oral PrEP group)

“We get educated here at the facility that the side effects will last for seven days. If seven days have passed and you are still experiencing side effects, you can come back to the facility…” (Gqeberha, oral PrEP group)

Resources, including a demonstration ring, pelvic model and information pamphlets, supported choice counselling and illustration ring insertion. Participants found these helpful in their decision‐making and practical use of the ring.

“It (pamphlet) was useful and easy to read for me… the first time I inserted it by myself I got the information from there (the pamphlet) on how to do it…” (Gqeberha, Ring group)

- **Learning about other PrEP options**

Participants learned about the ring while accessing oral PrEP refills or contraception services and highlighted the benefits of having multiple PrEP options, allowing them to choose the method that best suits their needs and preferences.

“I think that since we are different people, one will experience this with the pill, another this with the ring, or the injection. No one will have an excuse that we are using one thing… people have options to choose from the pill, ring, and injection.” (Gqeberha, Ring group)

Some participants who use oral PrEP highlighted side effects as one of the reasons for PrEP discontinuation and were excited to learn about other PrEP methods to use to overcome these challenges.

“Because others they have side effects, as she said, with pills. They are able to choose what will work for them. They are able to switch and get a method that will work for them.” (Tshwane, Ring group)

- **Supportive**

Participants valued respectful and knowledgeable HCPs who offered clear information and hands‐on demonstrations of ring insertion, boosting their confidence and skills to self‐insert the ring.

“… she showed me; first time she inserted it, and then the next time when I came back I said this time I would try for myself because you showed me, okay, this is how you wrap it and then you insert inside, and then I did so then it gets inserted nicely.” (Tshwane, Ring group)

While participants understood they could self‐insert the ring, they emphasized the value of having supportive HCPs available to assist when needed.

“She (nurse) asked if I will be able to do it (self‐insert the ring), I said if there is a booklet that explains everything, I will be able to do it, but if I get confused, I will come to her.” (Gqeberha, Ring group)

- **Privacy**

Participants appreciated confidentiality and privacy during consultations, despite the clinics being busy, making them to feel comfortable discussing their health concerns.

“Yes (there was privacy), because it's just the two of you in the room. She would also ask if everything were clear, or I need clarity so that she can explain again.” (Tshwane, oral PrEP group)

Although many participants shared positive experiences about choice counselling, some reported different experiences.

- **Not being provided with enough information**

Some participants felt that they received insufficient information about the ring and were not shown a demonstration ring or pelvic model. This resulted in uncertainty regarding the ring's size, appearance and insertion.

“I feel like they must tell us more about the ring. Maybe some of us are not even aware how big it is… So, if they can explain how it looks, what is the size, and how it works. Because for me, when I think about the ring, I think about something uncomfortable, and made of coil/copper… They have never showed us (the ring)…” (Mthatha, oral PrEP group)

- **Ring insertion provided by male HCPs**

Some participants felt uncomfortable with male HCPs inserting the ring or self‐inserting it in their presence, preferring to insert it themselves at home after receiving information on how to do so.

“He said he's trained to insert the ring, but I told him no, I rather take oral PrEP, never. Then he showed me and said I must insert it in front of him. I said, no, I will insert it myself at home, just explain to me how to do it…” (Gqeberha, Ring group)

Translation of guidance on ring insertion into vernacular languages at times led to misinterpretation, particularly if these had unintended sexual connotations, influencing how information was received.

“I wasn't comfortable because he kept on saying is it inserted? Is it inserted nicely. So, I kept on saying hmm (yes). So, it made me feel like (chuckles)” (incomplete statement) [the participant felt shy as she shared this experience with the male provider] (Tshwane, Ring group)

- **Lack of sufficient information and guidance on ring insertion**

Some participants who opted for oral PrEP felt that they received insufficient information about the ring and were not shown a demonstration ring or pelvic model. This resulted in uncertainty regarding the ring's size, appearance and insertion.

“I feel like they must tell us more about the ring. Maybe some of us are not even aware how big it is… So, if they can explain how it looks, what is the size, and how it works. Because for me, when I think about the ring, I think about something uncomfortable, and made of coil/copper… They have never showed us (the ring)…” (Mthatha, oral PrEP group)

Some participants who were assisted by an HCP to insert the ring during initiation felt uneasy with being provided rings for self‐insertion at follow‐up visits, as they were not confident in their ability to insert the ring correctly.

“The nurse inserted it the first [time]. The second time, that older lady (nurse) said she will insert it again. When I got here the third time, there was another lady (nurse), she gave me few rings to insert at home and I didn't know how to [insert].” (Mthatha, Ring group)

Encountering different HCPs during subsequent clinic visits led to assumptions that returning clients already knew how to insert the ring and providing multiple rings to self‐insert at home. In some instances, this led to ring discontinuation.

“…at the clinic you are just given the ring to insert yourself, you don't even know how to insert it. I was shown once and then I was given a lot of rings, then I decided that I'm not coming back I'd rather stay at home” (Gqeberha, Ring group)

- **Use of scientific jargon**

Participants expressed their dislike of words they did not understand as it hindered their understanding.

“Like the way the lady said that most of the times they explain to her using scientific words. And some words are too big, are bombastic, so are difficult for us to understand.” (Tshwane, Ring group)

### 3.2 Factors influencing PrEP method choice

**Figure 1:**
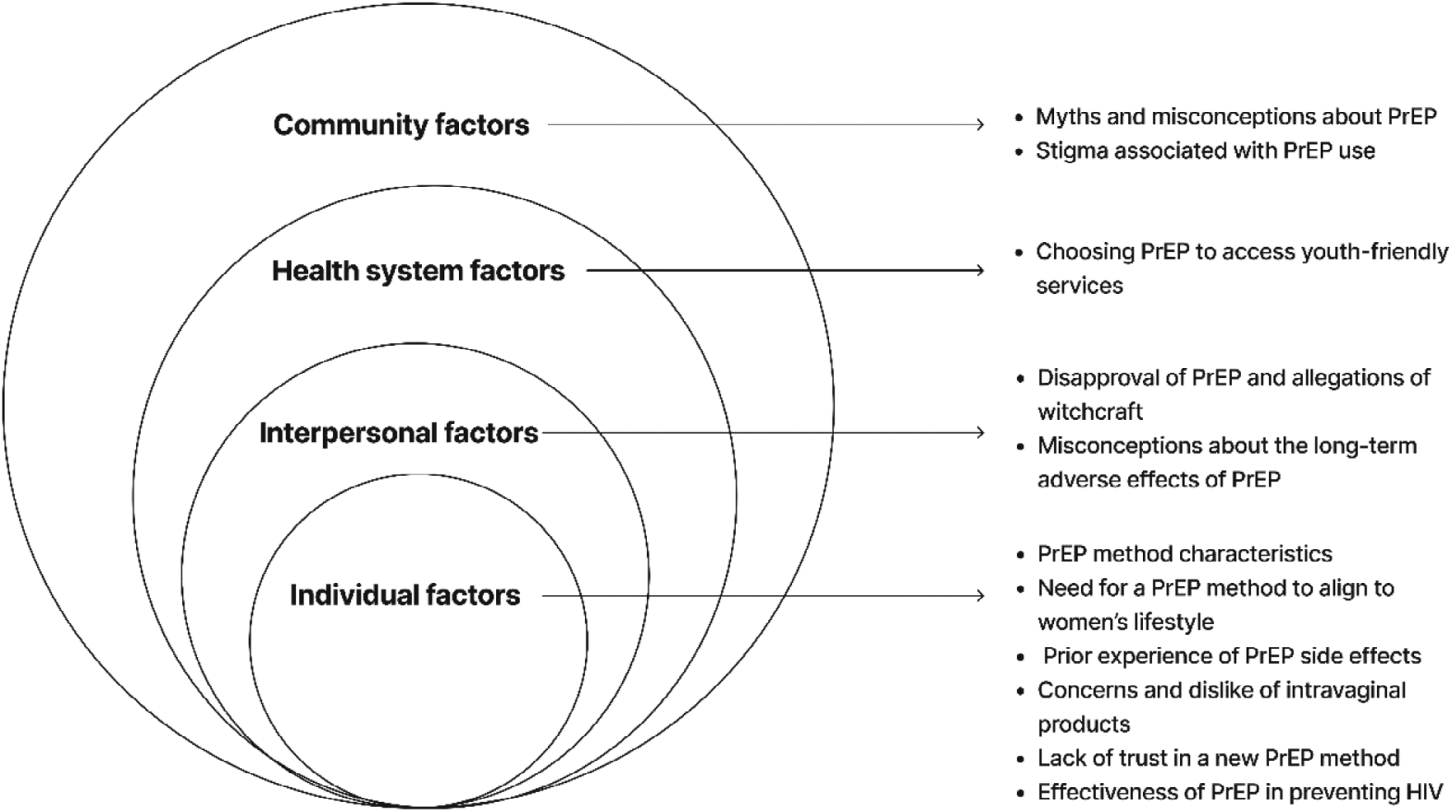
**Socio‐ecological model of factors influencing pre‐exposure prophylaxis (PrEP) method choice among women provided a choice of oral or ring PrEP through Project PrEP in South Africa**.

Participants described a range of influences of their PrEP method choice, which are presented according to four SEM constructs (Figure 1).

### 3.3 Individual‐level factors

- **PrEP method characteristics**

Participants’ choices were shaped by preferences about method‐specific characteristics. The oral PrEP was perceived as large and difficult to swallow, leading some participants to opt for the ring.

“…the struggle with swallowing and big pills was serious… when the ring was introduced, and they explained to me I decided to switch to it.” (Tshwane, Ring group)

Some participants preferred a long‐acting method and chose the ring, which was seen as addressing some of the challenges of daily pill taking, such as forgetfulness.

“I changed to the ring because I used to forget to take oral PrEP… Then I decided to switch to the PrEP ring because it's inside for 28 days…” (Mthatha, oral PrEP group)

Oral PrEP was chosen by those concerned that the ring, being locally acting, does not protect against HIV transmission through oral or anal sex, or accidental contact with blood containing HIV.

“She explained that you must keep it inside the vagina for 28 days… but it provides protection only for vaginal sex. That's one of the reasons I didn't like it.” (Mthatha, oral PrEP group)

- **Need for a PrEP method to align to women's lifestyles**

Many participants reported partying on weekends and alcohol consumption, leading to missed doses of oral PrEP due to forgetfulness or unavailability. They preferred the ring, finding it more compatible with their lifestyles as it remains in place and does not require daily dosing.

“The ring treats me well because I'm a someone who like to have fun, and drinks (alcohol), so it helps because I don't worry about forgetting, unlike the pills.” (Gqeberha, Ring group)

- **Prior experience of PrEP side effects**

Participants who had experienced side effects from oral PrEP, including dizziness, vomiting, nausea, tiredness and decreased appetite, chose the ring—perceiving it to have fewer or more tolerable side effects.

“Pills were making me asleep, so the side effects were too much. So, I decided to take ring because its side effects are fine. They are not painful, they are the same as period pains you can tolerate them…” (Tshwane, Ring group)

- **Concerns and dislike of intravaginal products**

Some participants expressed dislike and discomfort with inserting objects into the vagina, viewing it as taboo and believing only a penis should go inside the vagina. They also worried about incorrect insertion, physical discomfort, dislodgement during sex and accidental expulsion while walking or sitting.

“I don't feel I would be comfortable walking with it inside, I'd be worried that it will fall. There's only one thing (meaning penis) that need to go in there (laughs), not the ring…” (Mthatha, oral PrEP group)

“Or it might not sit correctly. I am not sure if I inserted it correctly or scared that maybe it might move during intercourse when he's penetrating…” (Tshwane, oral PrEP group)

Participants also expressed less confidence in an intravaginal method, being less familiar with this method than with pills which are more widely available, and used for numerous other medical indications, as one participant explained:

“…You see a pill is something that we know that when you have headache you can take it and get cured. So, with the ring I'm not sure if it will work for me or not…” (Tshwane, oral PrEP group)

- **Lack of trust in a new PrEP method**

Some participants expressed a lack of trust in the ring because it was new, preferring to continue with a PrEP method with which they had built familiarity and confidence.

“It (oral PrEP) is the only method I trust compared to others. I trust it and I'm also afraid of the vaginal ring. I am happy with the oral PrEP. I rather swallow the pills than use the ring.” (Mthatha, oral PrEP group)

They further highlighted the contribution of unknown side effects to this fear and mistrust.

“I think what scares the most of us is not knowing the side effects. Otherwise, if there was no oral PrEP, but just the ring we would take it…” (Gqeberha, oral PrEP group)

However, sharing testimonials from women who have successfully used the new method could overcome this:

“…Someone is telling me that it (ring) is indeed working but then we do not know if they are using it or not. So at least if there's an example from one person to show us that she is using it and then I will see after those months…” (Tshwane, oral PrEP group)

- **Effectiveness of PrEP in preventing HIV**

Although participants acknowledged the benefit of the ring in eliminating the burden of daily pill taking, concerns about its efficacy influenced their choice.

“I didn't have a problem with the ring because it's better than taking oral PrEP every day, but my concern was it's only 35% effective.” (Gqeberha, oral PrEP group)

### 3.4 Interpersonal‐level factors

- **Disapproval of PrEP and allegations of witchcraft**

Participants feared disclosing their ring use to partners, reporting they had complained of feeling the ring during sex or expressed concerns it would affect their sexual performance.

“My husband asked if I have a snake in my vagina… he felt it and asked what is this that I keep bumping on it? Is it a snake? I said, no, it's not a snake, it's a ring. He said, no, remove this thing, I want to put my whistle (meaning his penis). I had to be obedient and remove it… Then I thought I must switch back to the pill.” (Mthatha, Ring group)

One participant discussed her decision to use the ring with her partner, but his disapproval resulted in her choosing oral PrEP.

“I didn't have a problem with the ring the problem was my partner… I told him that there's a ring and I have decided to take it. He asked if it would not affect our sexual activity… he said no, I can't take it because it's inside the vagina and maybe he will feel it when we have sex…” (Gqeberha, oral PrEP group)

Due to cultural taboos against inserting objects into the vagina, partners associated the ring with witchcraft.

“…you know people have beliefs… Once someone feels that there's something in you, he will think that you want to trap him, you want him to be with you forever… he just took it out…” (Gqeberha, Ring group)

**(ii) Misconceptions about the long‐term adverse effects of PrEP**

Some participants faced judgemental attitudes from families, who mistakenly believed that the ring could lead to adverse effects later in life.

“My mom said that it shocked her because it was her first time seeing it with me, and she said, ‘No, [participant's name is omitted] it is better to go back to pills’… it scares her because she's thinking that it might cause kidney failure and I thought what if she's right, and then I switched again…” (Tshwane, oral PrEP group)

### 3.5 Health system–level factors

**(i) Choosing PrEP to access youth‐friendly services**

At study sites, rings are provided via youth‐friendly services, with shorter waiting times and a welcoming environment. Older clients feared being transferred to other service points and opted for the ring, perceiving it to be more accessible.

“Another thing is the issue of age because they said if you're over the age of 25, I think, you must go inside the clinic and you must queue where everyone else is queuing as if you are sick. So, I had to switch to the ring.” (Gqeberha, Ring group)

### 3.6 Community‐level factors

Myths, misconceptions and stigma associated with PrEP use within communities also influenced women's PrEP choice.

- **Myths and misconceptions about PrEP**

Some participants reported how hearing negative beliefs or experiences of PrEP, influenced their choice.

“…people say negative things about the pill. They say it increases pressure and has a lot of side effects. I came to initiate [oral] PrEP… But I didn't use it because I'm scared of using it. [Client navigator's name omitted] told me that there's a new available method and I started to use the ring.” (Mthatha, Ring group)

One participant avoided the ring after she heard a woman expressing discomfort with its use.

“There was a lady in the queue who had a ring and she said she had come to take it out because it's painful, she can't even walk properly. By the time I went inside to see the nurse, I knew already that I was not going to choose the ring.” (Mthatha, oral PrEP group)

- **Stigma associated with PrEP use**

HIV‐related stigma significantly influenced participants’ method choice, with a fear of others assuming they are living with HIV because of taking oral PrEP.

“…I don't like people see me taking pills. I become a bit embarrassed because they would say this one is sick.” (Tshwane, Ring group)

Adopting HIV‐related stigma within the community also influenced their method choice.

“That's what made me take it (ring), because I heard about oral thing (PrEP) here at the clinic initially, and I said, I will never take pills while I'm not sick…” (Gqeberha, Ring group)

## 4 DISCUSSION

This study presents some of the first experiences of women accessing PrEP choice counselling and the factors influencing their method choice in primary care settings in South Africa. Women's experiences of choice counselling were largely positive, appreciating accessible information and practical demonstrations. Women valued supportive HCPs and privacy during counselling but expressed discomfort with male providers and wanted more details on side effects and guidance on ring insertion. Influences of PrEP choice included method efficacy, hesitancy around intravaginal methods, lack of trust in new methods, prior experiences with oral PrEP and the need for methods to suit women's lifestyles. The perceptions of sexual partners, families and the community played an important role in women's PrEP choice.

Comprehensive, non‐technical information and individualized counselling, empowered women to make informed PrEP choices. Demonstration of ring insertion and supportive guidance from HCPs equipped women with skills for ring self‐insertion, although some expressed the need for ongoing guidance and support. This highlights how initial fears of ring self‐insertion reported elsewhere [21], may be overcome through sensitive guidance and reassurance.

Women's discomfort with male HCPs aligns with findings from studies on intrauterine devices or vaginal examinations [22, 23]. In this study, male HCP's use of vernacular words during ring demonstrations carried unintentional sexual connotations, which may have contributed to this experience. While support during ring insertion is crucial, male HCPs should be sensitized to women's concerns and not overlook preferences for self‐insertion. Additionally, culturally and gender‐sensitive language should be considered when translating information.

Women in our study had varied preferences for PrEP methods. Negative experiences with oral PrEP often drove a preference for the ring. However, many women chose oral PrEP for its higher efficacy, consistent with findings from other studies [21, 24, 25, 26]. Despite the high acceptability of the ring in clinical trials [10, 27, 28], mistrust of a new product and dislike of intravaginal products influenced its acceptability in our setting. In our context, intravaginal products such as female condoms and intravaginal contraceptives are not widely used, due to cultural taboos and negative beliefs [21, 29, 30]. Our relatively young study population may have contributed to this finding, given reports of a stronger dislike for vaginally administered methods among younger women [28]. The introduction of a new product is complex, requiring action at multiple levels, including community interventions to address myths and misconceptions [13]. Appropriate information on ring safety and culturally acceptable messages about ring insertion will be crucial to future introduction. Providing women an opportunity to try the ring and obtaining positive testimonies from women who have used it could improve familiarity, dispel fears and misconceptions and possibly increase uptake [28, 31].

Partners’ fears and negative perceptions of the ring strongly influenced women's PrEP choice, reflecting a gap in male partner's knowledge about the ring and consistent with studies reporting partner disapproval of ring use by women [32, 33, 34]. Involving male partners in education, decision‐making and addressing their concerns can overcome these challenges, while maintaining trust in relationships and supporting female‐initiated methods [33, 35, 36]. Strategies like this could address partners’ negative perceptions of the ring reported in this study.

Although our research did not fully explore the contribution of gender dynamics to PrEP choice, studies have noted the influence of gender dynamics and relationship power imbalances on PrEP use and adherence [37, 38]. In addition to partners, judgemental attitudes and misconceptions about the ring from family members influenced women's choice. Families often play an important role in supporting PrEP use [39]; however, in this study, they instilled fear and discouraged participants from using the ring. Empowering women to provide information about the ring with families and partners is essential to increase support. Similarly, community stigma around oral PrEP, misconceptions about the ring and perceived experiences of other women using PrEP influenced women's PrEP choice. Studies of young women in South Africa and Uganda found that communities can be highly influential in women's decisions to use vaginal rings [20, 40, 41], highlighting the need for community engagement to identify and dispel myths, and create demand for PrEP services as new PrEP methods are introduced [13]. The overarching desire for women to access confidential, private and respectful health services was highlighted in our findings. Addressing barriers such as long queues and lack of privacy [42, 43], is crucial for effective PrEP service delivery, regardless of the methods offered.

### 4.1 Limitations

We acknowledge the risk of social desirability bias in this study, as FGDs were conducted at sites where participants accessed health services, therefore, participants might have provided favourable responses. We sought to capture diverse experiences without aiming for saturation in any single aspect. We acknowledge that this approach may have led to a less comprehensive understanding of specific themes within the data, and the diverse experiences identified may not be transferable to similar populations. Partners influenced women's decision‐making; however, their views were not sought in this study. Future research is needed to explore partner's perspectives on women's use of the ring in our setting. Our study only focused on oral PrEP and the ring. Future research will need to explore how women's choices evolve with the introduction of additional PrEP methods.

## 5 CONCLUSIONS

Women's experiences of choice counselling were largely positive, reiterating the benefits of having PrEP choice, and emphasizing the need for competent, supportive HCPs and accessible information to support decision‐making. Multiple influences on PrEP method choice highlight the need for engagement of communities, partners and families to support women in this process. Tailored, culturally acceptable messages about the ring's insertion and obtaining testimonies from women who have used the ring could enhance demand and acceptability in our context.

## COMPETING INTERESTS

The authors have no conflicts of interest to declare.

## AUTHORS’ CONTRIBUTIONS

CEM and SM contributed to the conception and design of the study. FAC, FMM, SD and AK reviewed the transcripts. FAC and FMM coded and analysed the data. SD wrote the first draft of the manuscript. All authors reviewed the manuscript, contributed to the revision and approved the submitted version.

## FUNDING

This work was supported by Unitaid [grant number 2017‐21‐Wits‐PrEP].

## Supporting information




**Appendix S1**: COREQ checklist

## Data Availability

The data that support the findings of this study are available from the corresponding author upon request.
