# Supplementary material for: “People have options”: a qualitative study of experiences and influences of PrEP choice among women in South Africa
Source: J Int AIDS Soc. 2025 Jul 2;28(Suppl 2):e26462. doi: 10.1002/jia2.26462 (PMC12215813; doi:10.1002/jia2.26462)
Supplement: Supplementary file 1 — Appendix S1: COREQ checklist [file JIA2-28-e26462-s001.pdf]

APPENDIX 1 - COREQ checklist

The Consolidated Criteria for Reporting Qualitative Studies (COREQ): 32-item checklist

Item Guide questions/description Notes	Item Guide questions/description Notes	Item Guide questions/description Notes
Domain 1: Research team and reflexivity		
The research team<br>The research team was multidisciplinary, bringing together expertise from various fields. It included professionals with backgrounds in public health, medicine, psychology and social sciences. Team members held advanced degrees such as PhDs, master's degrees, and medical degree, including MBChB with specializations in tropical medicine and HIV management. This diverse expertise ensured a well-rounded approach to the study.		
Personal Characteristics		
1. Interviewer/facilitator	Which author/s conducted the interview or focus group?	FAC and SD conducted focus group discussions.
2. Credentials	What were the researcher's credentials? E.g. PhD, MD	FAC: MA<br>SD: PhD
3. Occupation	What was their occupation at the time of the study?	FAC: Associate Researcher<br>SD: Senior Researcher
4. Gender	Was the researcher male or female?	All focus group discussion facilitators were female (FAC) and (SD.)
5. Experience and training	What experience or training did the researcher(s) have?	FAC and SD have experience in qualitative research methods including facilitating focus groups and conducting in-depth interviews.
Relationship with participants		
6. Relationship established	Was a relationship established prior to study commencement?	Participants were informed about the purpose of the discussion before data collection, which was covered in the ICF. Additionally, before the discussion, the facilitators reiterated the purpose of the discussion to the participants.
7. Participant knowledge of the interviewer	What did the participants know about the researcher? e.g. personal goals, reasons for doing the research	Participants were aware that this was a research project to explore PrEP use and choice.

Developed from:
(Tong, A., et al. (2007). "Consolidated criteria for reporting qualitative research (COREQ): a 32-item checklist for interviews and focus groups." International Journal for Quality in Health Care **19**(6): 349-357.

8. Interviewer characteristics	What characteristics were reported about the inter viewer/facilitator?<br>e.g. Bias, assumptions, reasons and interests in the research topic	Researcher reflexivity was crucial in the FGDs conducted by two female researchers on PrEP use and method choice among women users. Their shared gender identity fostered a comfortable environment, encouraging open discussions about experiences, and preferences. We recognize that researchers' professional training and experience may influence interpretation of participants' perspectives, however we employed phenomenological approach in our study to prioritize participants' voices.
Domain 2: Study design		
Theoretical framework		
9. Methodological orientation and Theory	What methodological orientation was stated to underpin the study? e.g. grounded theory, discourse analysis, ethnography, phenomenology, content analysis	Our study employed a phenomenological and exploratory approach utilizing focus group discussions.<br>We analysed the data in an inductive manner using the Socio-Ecological Model (SEM) framework to guide our analysis. The SEM framework was applied to explore factors influencing women's PrEP method choice. Women's experiences with PrEP counselling were grouped into positive and negative experiences. However, the societal level was not explored because factors influencing PrEP choice at this level did not emerge from the data.
10. Sampling	How were participants selected? e.g. purposive, convenience, consecutive, snowball	Participants were purposively selected. We grouped participants according to the PrEP method they were using or had used.
11. Method of approach	How were participants approached?<br>e.g. face-to-face, telephone, mail, email	Recruitment was conducted by site-based fieldworkers during clinic visits, or telephonically.

Developed from:
(Tong, A., et al. (2007). "Consolidated criteria for reporting qualitative research (COREQ): a 32-item checklist for interviews and focus groups." International Journal for Quality in Health Care **19**(6): 349-357.

12. Sample size	How many participants were in the study?	126 women participated in the study.
13. Non-participation	How many people refused to participate or dropped out?<br>Reasons?	189 participants were screened for eligibility, 182 were eligible and 126 agreed to participate in the study. Data for all 126 participants were included in the final analysis.<br>Reasons for non-participation included eligible participants being unavailable or not picking up reminder calls on the day scheduled for FGDs.
Setting		
14. Setting of data collection	Where was the data collected? e.g. home, clinic, workplace	Focus group discussions were conducted in a private room either at the clinic facility or a nearby community-based organization.
15. Presence of nonparticipants	Was anyone else present besides the participants and researchers?	There were no people present during the data collection besides participants and researchers.
16. Description of sample	What are the important characteristics of the sample? e.g. demographic data, date	Of the 126 participants, the majority (61.1%) were <25 years, almost all were in a relationship (92.1%), the majority had completed high school (62.7%) and 80.9% were unemployed.
Data collection		
17. Interview guide	Were questions, prompts, guides provided by the authors? Was it pilot tested?	A semi-structured guide was used to direct the discussion.<br>The semi-structured guide was not piloted
18. Repeat interviews	Were repeat inter views carried out?<br>If yes, how many?	There were no repeat interviews. It was a once-off focus group discussion.
19. Audio/visual recording	Did the research use audio or visual recording to collect the data?	All interviews were audio recorded with the permission of participants.
20. Field notes	Were field notes made during and/or after the interview or focus group?	Researchers made field notes during the focus group discussions.
21. Duration	What was the duration of the interviews or focus group?	The focus groups lasted 59 minutes to 2 hours.

Developed from:
(Tong, A., et al. (2007). "Consolidated criteria for reporting qualitative research (COREQ): a 32-item checklist for interviews and focus groups." International Journal for Quality in Health Care **19**(6): 349-357.

22. Data saturation	Was data saturation discussed?	We focused on capturing diverse perspectives, which resulted in a broad range of experiences rather than reaching data saturation on a single aspect.
23. Transcripts returned	Were transcripts returned to participants for comment and/or correction?	Transcripts were not returned to participants for comment or correction.
Domain 3: Analysis and findings		
Data analysis		
24. Number of data coders	How many data coders coded the data?	Two researchers (FAC and FMM) independently coded three transcripts using open coding, compared codes to ensure consistency and developed a preliminary coding framework. This was reviewed by the team, revised and the coding framework finalized, after which the remaining transcripts were analysed.
25. Description of the coding tree	Did authors provide a description of the coding tree?	There is no description of the coding tree.
26. Derivation of themes	Were themes identified in advance or derived from the data?	Themes were derived from the data in an inductive manner.
27. Software	What software, if applicable, was used to manage the data?	Researchers used NVivo version 14 to code all interviews.
28. Participant checking	Did participants provide feedback on the findings?	Participants did not provide feedback on the findings.
Reporting		
29. Quotations presented	Were participant quotations presented to illustrate the themes/ findings? Was each quotation identified? E.g. participant number	Key findings of the study were reported with selected quotes in text.
30. Data and findings consistent	Was there consistency between the data presented and the findings?	All findings were derived from the data and all themes are supported by illustrative quotes.

Developed from:
(Tong, A., et al. (2007). "Consolidated criteria for reporting qualitative research (COREQ): a 32-item checklist for interviews and focus groups." International Journal for Quality in Health Care **19**(6): 349-357.

31. Clarity of major themes	Were major themes clearly presented in the findings?	Major themes are clearly defined by a paragraph title.
32. Clarity of minor themes	Is there a description of diverse cases or discussion of minor themes?	Findings of our study includes both major and minor themes that emerged on the data. During the analysis, we captured a range of participant perspectives to ensure understanding of their experiences of PrEP choice, and the factors influencing PrEP choice.

Developed from:
(Tong, A., et al. (2007). "Consolidated criteria for reporting qualitative research (COREQ): a 32-item checklist for interviews and focus groups." International Journal for Quality in Health Care **19**(6): 349-357.
